# Well-being and internal resources during the COVID-19 pandemic in relation to meaning in life and existential anxiety

**DOI:** 10.3389/fpsyg.2023.1168641

**Published:** 2023-08-25

**Authors:** Laura Teodora David, Camelia Truţa

**Affiliations:** Faculty of Psychology and Education Sciences, Transilvania University of Braşov, Braşov, Romania

**Keywords:** meaning in life, existential anxiety, well-being, creativity, playfulness, COVID-19

## Abstract

The COVID-19 pandemic represents an event that placed humanity in a context where confrontation with uncertainty, isolation, life threats, and significant changes in one's life were on a scale that exceeded by far any previous individual or community crises. The interest of the present research was to investigate the relationship between meaning in life (MiL) and existential anxiety (EA) with personal internal resources such as creativity, playfulness, well-being, and personal meaning. A total of 451 participants from 48 countries (mean age 34.93 years, standard deviation 12.62, 31.9% men, 67.4% women) were questioned via online questionnaires between May and June 2020. Cluster analysis was performed on the meaning in life and existential anxiety that generated four categories of persons: Reactive, Superficial, Preoccupied, and Dedicated. Well-being and internal resources were associated mostly with the Dedicated type and less with the Reactive one. Arguments relying on the existential positive psychology suffering model and the hostile world scenario are presented to support the results and plead for interventions that elicit meaning, stimulate creativity, and guide people in finding purpose in order to ultimately promote psychological and mental health.

## 1. Introduction

Life was disrupted worldwide in the spring of 2020 when the coronavirus spread far enough to threaten the health and lives of humans across the globe. Lockdown was decided by many governments, and social distancing, wearing masks, and multiple hygienic measures were also strongly imposed. The COVID-19 pandemic was declared, and people were confronted with an unknown situation. The novelty was not accompanied by excitement or joy, quite the opposite, it induced uncertainty, fear, and isolation. Life was changed from 1 day to another; new habits and new routines had to be formed and a chain of effects started to unfold. Consequences intruded every aspect of life: physical, social, professional, and economic (WHO, 2022, Impact-of-COVID-19-on-people; Lekagul et al., 2022), but the effects were at the same time general and country specific. More than 700 million people tested positive and more than 6.5 million people died (as of December 2022 WHO statistics), numbers that support the extension COVID-19 pandemic had global and personal consequences.

On a personal level, the COVID-19 pandemic felt like a veritable existential crisis, implying isolation, lack of freedom, the salience of death and loss, and a gradually diminished sense of comprehension while chaos and uncertain feelings and thoughts developed (Van Tongeren and Showalter Van Tongeren, 2021). It activated existential questions about life and the meaning of life and favored existential anxiety.

The present study focuses on how the two constructs, meaning in life and existential anxiety, coexisted in times of predicament and their associations with inner resources such as psychological well-being (eudaimonic side), creative potential, playfulness, and personal meaning profile. A review of the constructs is presented below.

Meaning in life (MiL) has long been an interesting topic, discussed and explored from more than one angle. The most down-to-earth denotation of meaning is the lexical one—to make sense, identify patterns with some kind of significance, and make connections between events and situations in order to understand them. And with this—the understanding part, the essence of meaning becomes more philosophical, relates to awareness, to our need to see existence as fit and worthwhile. Kierkegaard and Nietzsche, existentialist philosophers, were both concerned with what brings meaning to life—transcendent values or living one's life with authenticity and power to make your own decision. There were even questions about the most suitable phrase: meaning *of* life (like in what is the point of existence) or meaning *in* life (what gives significance and value in someone's life). In psychology, the appeal of the construct is connected with attention given to Frankl's theory of man in search for meaning and grew in several directions: cognitive, motivational, and affective perspectives (Batthyany and Russo-Netzer, 2014; Martela and Steger, 2016). Eagelton (2007) differentiates between the meaning of existence and the meaning of life, with the last construct underlying the self-reflection on the degree of fulfillment in life, closer to the psychological perspective, and the first one closer to the philosophical understanding.

From the cognitive perspective, meaning is seen as a mental representation that everything is coherent and makes sense: the world and life have stability, coherence, and consistency and offer fulfillment (Baumeister, 1991). Another perspective on meaning in life underlies its motivational function. Meaning is seen as establishing purpose, goals that can be reached or at least pursued, in order to flourish and be productive. Meaning in life also includes an affective dimension, which is linked to satisfaction and happiness and contentment with one's life.

A recent framework for meaning in life concentrates on three dimensions of the construct: purpose, coherence, and significance (Martela and Steger, 2016). The purpose was sometimes considered synonymous with meaning in life, involving the setting of long- and short-time goals and taking action in order to accomplish these goals. Coherence implies the belief that life is predictable and makes sense and that it fits into a broader context (interpersonal, societal, historical, or cultural). In other words, this ability to understand the environment and to recognize patterns is a cognitive experience that contributes to the meaning in life (MacKenzie and Baumeister, 2014). It has a descriptive nature and supports the capacity to construct mental models of the world. Significance is associated with a life worth living, that has value. It is also considered an evaluation made by individuals regarding the mattering of their life in the world, not only for their own consideration but as valuable individuals in the eyes of others (George and Park, 2014). Significance and purpose have an evaluative nature and confer positive or negative connotations of events or life, the sense of right or wrong, directing or blocking future actions (Crescioni and Baumeister, 2013). The three dimensions are entangled with coherence being necessary for finding significance, purpose also contributing to significance, and significance employing motivation in life, aka purpose (Martela and Steger, 2016).

Meaning in life is seen also dichotomous—as the presence of meaning or search for meaning (Steger et al., 2011). People with high presence and high search are more satisfied with their life compared with any other category, and those with low presence and low search are the most dissatisfied with their life.

Wong (2010) makes an interesting proposal for bringing together the existential viewpoints of the meaning of life with the positive perspective which was referred to as existential positive psychology. This standpoint combines the focus on human strength with a focus on the finitude of the human condition in the face of death to explore the potential of identity crises or discontent in life for cultivating authenticity and happiness. The quest for purpose generates meaning, and death anxiety allows self-transcendence. Wong proposes the PURE model to understand meaning as four components: Purpose, Understanding the demand of a situation, taking Responsible action, and Evaluating the actions in order to assure authenticity. Exploring more on the existential and positive perspective, Van Tongeren and Showalter Van Tongeren (2021) describe the existential positive psychology suffering model (EPPSM) where suffering, as a life component, disturbs beliefs about the world, and it is chronic and implies profound consequences. When suffering occurs, it also brings existential anxiety that impairs the ability to understand life and give meaning to existence, losing indicators of a positive perspective on life. To overcome suffering, efforts should be made to elicit meaning that will mitigate existential anxiety.

The attention given to meaning in life also has to do with its role in interaction with other factors that explain behavior or emotional outcomes in people. Aversive contexts that life brings are studied in connection with meaning in life seen as a reservoir of strengths, beliefs, values, and goals. The content of the reservoir inclines to change and varies or remains stable and protects a person's well-being (Martela and Steger, 2016; Seidel et al., 2023). Feeling lonely and not able to control and decide for yourself can yield diminished meaning in life (Kim et al., 2014). The social isolation imposed by the COVID-19 pandemic in the early months of 2020 increased loneliness as it interfered with one of the main sources of meaning and well-being, namely with individuals' significant social relationships. On the other hand, life purposes and values may contribute to overcoming difficulties (Wong, 2010; McDonald et al., 2012; Kim et al., 2014). Personal and professional goals, alongside social responsibility, contribute to finding meaning in life (Bhattacharya, 2011).

One interaction particularly studied was the relationship between the meaning in life and well-being, as well as a multidimensional construct that is not reduced to happiness and positive affect. Diener (1984) defines well-being as a subjective construct that resides within the experience of the individual (p. 543), is a positive integrated judgment of a person's life (p. 544), and is not only the absence of negative affect. This approach is closer to the hedonic view of well-being, which includes also life satisfaction (Diener et al., 1985). Psychological well-being (Ryff and Keyes, 1995; Ryff et al., 2004) is a multifaceted concept, seen as the eudaimonic model of well-being that is not a trait-like dimension, but a dynamic one, depending on life events and transitions.

Smotkin and Shira (2013) propose two models of interaction between meaning in life and subjective well-being: the amplification model, with both variables at high levels, which sustain mobilization of internal resources (SWB and MiL will overlap), and the compensation model (when one variable is low, the other will increase in order to balance the needs of the person). Both variables interact with each other, high meaning in life and subjective well-being favor adaptive behaviors. The same authors introduce the hostile world scenario model (HWS, Shmotkin, 2011; Smotkin and Shira, 2013), which represents a system of appraisal that a person activates to detect potential or real threats to physical or mental integrity. When confronted with danger (COVID-19 pandemic corresponds to HWS event), meaning in life supports construction/reconstruction of life by interpreting/reinterpreting life adversities and has a protective role, while subjective well-being supports positive appraisal of life even in negative conditions, so crises become manageable and anxiety regulated.

Difficult times such as the recent pandemic elicit existential anxiety (Popovic, 2002; Weems et al., 2004) consisting of unresting feelings about the future, the world, and self-existence. It involves fear of death, feelings of emptiness and lack of meaning, guilt, and self-condemnation. Existential anxiety manifests when people become overwhelmed by the awareness that fate is circumstantial and death is unavoidable, when their beliefs are no longer valid or important and rules and principles that governed their actions make no sense anymore and when there is a sense that the life they lived did not meet the expectation of a good life or did not satisfy personal or universal standards. It builds up in times of uncertainty, insecurity, and isolation, “the perfect storm” during the COVID-19 quarantine. A way to face adversity is to focus on meaning in life, at both a micro level (terrestrial meaning) and macro level (cosmic meaning). Meaning in life represents a buffer of existential anxiety, helping people tolerate or cope with the hardship of existence and the awareness of one's mortality (Kesebir and Pyszczynski, 2014). Is the sense of understanding and believing that “life and death exist simultaneously, not consecutively” as Yalom clearly puts it (Yalom, 1980, p. 29).

When confronted with adversity, internal strengths such as creativity or playfulness as resources may contribute to and associate with higher meaning in life and lower pathology. Creativity is defined as underlying at least two simultaneous characteristics: originality and value/usefulness (Lubart and Guignard, 2004; Runco and Jaeger, 2012). It manifests as Big C when the impact of creativity is on a societal level, or as little c—displayed at an individual level translating into the ability to solve everyday challenges in novel ways. Little c manifests spontaneously or after a time of preparation and commitment (Richards, 2007). Furthermore, creativity becomes a resource to find meaning by expressing yourself and gaining some control in the face of existence coercions. Creativity gives an individual freedom and a way to master the situation as Cozzolino and Blackie (2013) included it in the specific—existential system which activates positive features of a person (Cozzolino and Blackie, 2013).

The role creativity plays in confrontation with crises that activate existential anxiety is explained by the terror management theory (TMT, Greenberg et al., 1986). This model implies that being aware of one's own mortality and life's implacable end generates terror (anxiety). In order to manage anxiety and promote survival needs, a person activates beliefs and values that consolidate life meaning and even offer the possibility to transcend death. Combined with internal resources such as creativity, symbolic immortality can be achieved. As such, creative achievement was established as an anxiety-buffering mechanism, in the context of death awareness (Perach and Wisman, 2019). Death awareness is the same cognitive process that elevates existential anxiety.

Finally, another internal resource that helps a person to overcome difficulties in life and that we included in our study is playfulness. It is defined as “the predisposition to frame (or reframe) a situation in such a way as to provide oneself (and possibly others) with amusement, humor, and/or entertainment” (Barnett, 2007 p. 955). Playfulness was conceptualized in several studies as an inner strength, and playful adults are more inclined to follow inner goals instead of external ones and to be intuitive and ingenious (Proyer, 2012). Humphrey and Vari (2021) showed that intrinsic aspiration is a positive predictor of meaning in life, connecting in this way playfulness to meaning.

## 2. Research questions

The current study focuses on how people addressed the existential concerns activated by the COVID-19 pandemic. We founded our study on the assumption that the pandemic activated existential concerns and questions regarding one's own meaning in life which, in turn, affected general well-being and drove people to activate internal resources that helped them cope with the situation. As shown, several theoretical models and previous studies have tried to explain the relationship between existential anxiety, meaning in life, and different internal resources in confrontation with adverse life events indicating that adversity and threat to one's own life usually activate existential anxiety while meaning in life, well-being, and internal resources may act as buffers. From a positive psychology perspective, in this study, we conceptualized creativity, playfulness, and sources of personal meaning as internal resources. We were mostly interested in identifying the relationships between these variables in the specific context of the pandemic. Therefore, the main research questions of our study were as follows:

- How did existential anxiety concerns and people's meaning in life interact during the peak of COVID-19?
- What role did existential anxiety and meaning in life play in people's well-being?
- What were the significant differences in people's activation of internal resources (namely, creativity, playfulness, and personal meaning profile) in relation to existential anxiety and meaning in life?
- Were there any associations between people's changes in daily activities and consumption habits related to existential anxiety and meaning in life?

## 3. Methods

### 3.1. Participants and procedure

For this study, 451 participants filled in an online survey posted on social media or sent via email. The sample is a convenient one consisting of participants from all over the globe. The survey was posted on several social media international groups relating to the COVID-19 pandemic topics between May and June 2020, the peak of the first wave of the pandemic in Europe. Respondents come from 48 countries (mostly Europeans-−76.49%), with a mean age of 34.39 years (SD = 12.62). In total, 67.4% of the participants were women (31.9% were men, whereas 0.7% did not mention their sex). All participants completed the survey in English. We also collected data on several demographic characteristics such as level of education, employment status, relationship status, religion, and diagnosis of SARS-CoV-2 infection within their social network (Table 1).

**Table 1:** Participant demographics.

	**N (%)**		**%**
**Region**		**Relationship status**	
Europe	345 (76.49%)	Married	37.25%
US and Canada	62 (13.74%)	Single, never married	28.82%
Asia	35 (7.76%)	Single, divorced or widowed	3.55%
South America	6 (1.33%)	In a relationship, not married	27.27%
Africa	2 (.04%)	Prefer not to say	3.10%
Australia	1 (.02%)	**Employment status**	
**Residence**		Full-time employed	51.00%
In the country of citizenship	334 (74.05%)	Part-time employed	7.76%
Expats	117 (25.94%)	Self-employed	8.65%
**Level of education**		Not employed	3.77%
Less than high school degree	1.55%	Lost employment since the start of Covid-19 Pandemic	1.77%
High school degree or equivalent	13.97%	Retired	1.55%
Bachelor degree	33.04%	Student	23.50%
Master degree or equivalent	34.15%	Another situation	2.00%
PhD or higher	17.29%	**Infection with SARS-CoV2**	
**Religion**		Yes, myself	0.90%
Catholic	15.74%	Yes, a member of the family	4.28%
Orthodox	33.70%	Yes, a friend or acquaintance	35.14%
Islam	2.88%	I don't know anyone infected	59.68%
Other	18.40%		
Non-religious/ Atheism	24.39%		
Prefer not to say	4.88%		

### 3.2. Measures

Meaning in life was measured with the *Meaning in life questionnaire* (Steger et al., 2006). The 10-item scale assesses on a 5-point Likert scale the meaning in life as the sense and significance felt regarding one's own life and existence on two dimensions: the presence of meaning (Cronbach's alpha = 0.90) and searching of meaning (Cronbach's alpha = 0.90). The overall Cronbach's alpha coefficient for the scale was 0.78.

For measuring existential anxiety concerns, we used the *Existential anxiety questionnaire* (Weems et al., 2004), a 13-item measure on a 5-point Likert scale. The EAQ assesses anxiety concerns about fate and death (Cronbach's alpha = 0.68), anxiety about emptiness and meaningless (Cronbach's alpha = 0.68), and anxiety related to guilt and condemnation (Cronbach's alpha = 0.68). The overall Cronbach's alpha coefficient for the entire scale was 0.84 showing good internal consistency.

*Psychological Well-being* was measured using Ryff's scales (1995) comprising 18 items on a 7-point Likert scale grouped in five subscales: autonomy, environmental mastery, personal growth, positive relations with others, and purpose in life with Cronbach's alpha coefficients varying from 0.68 to 0.71. Cronbach's alpha coefficient for the overall scale was 0.81.

We conceptualized three internal resources in this study, creativity, playfulness, and personal meaning. *Creativity* as internal potential was measured with a 12-item questionnaire developed by Fürst and Grin (2018). It assesses the generation and selection of creative ideas in everyday work or leisure activities, on a 5-point Likert scale. The Cronbach's alpha coefficient for the scale was 0.89. Playfulness as an internal resource was measured with the *Short measure for adult playfulness* (Proyer, 2012). It comprises five items on a 5-point Likert scale assessing internal disposition to engage in playful activities and interactions, as a unidimensional measure. The Cronbach's alpha coefficient for the scale was 0.89. Finally, *Personal meaning profile* (McDonald et al., 2012) was used to assess the participants' perceptions of their own sources of a meaningful life. It comprises 21 items on a 7-point Likert scale measuring achievement, relationship, religion, self-transcendence, self-acceptance, intimacy, and fair treatment (with Cronbach's alpha coefficients between 0.63 and 0.88). The Cronbach's alpha coefficient for the overall scale was 0.85.

Additionally, the survey included several items designed to measure potential changes in everyday activities and consumption habits as a result of the COVID-19 pandemic. Participants were asked about: changes in the time allocated to work/academic tasks, cooking and cleaning, online and in-store shopping, volunteering, hobbies, family, watching TV/movies/streaming services, social media, online gaming, and time spent alone; and changes in their consumption of alcohol, non-alcoholic substances (such as nicotine or caffeine), psycho-active drugs, and psycho-active substances. For all these items, we used a nominal scale with the following response options: time/consumption decreased, time/consumption stayed the same, time/consumption increased, prefer not to say, and non-user.

## 4. Findings

The first step of data analysis revealed significant correlations between existential anxiety, meaning in life, and well-being. Creativity, playfulness, and personal meaning significantly correlate with meaning in life and well-being, while only personal meaning profile is associated with existential anxiety (Table 2). As expected, existential anxiety negatively correlates with meaning in life and well-being showing that individuals expressing more existential concerns tend to have lower levels of well-being and a decreased sense of meaning in their lives. Meaning in life positively and moderately correlates with well-being. Correlations also show that people with high levels of internal resources tend to report a greater presence of meaning and greater well-being.

**Table 2:** Correlation matrix between the investigated variables.

	**M**	**SD**	**1**	**2**	**3**	**4**	**5**	**6**
1. Existential anxiety	2.43	0.75	1					
2. Meaning in life	4.83	1.01	−0.16^**^	1				
3. Well-being	5.24	0.75	−0.53^**^	0.32^**^	1			
4. Creativity	3.55	0.68	−0.07	0.27^**^	0.36^**^	1		
5. Playfulness	3.53	0.92	−0.08	0.18^**^	0.28^**^	0.42^**^	1	
6. Personal meaning	5.06	0.81	−0.32^**^	0.55^**^	0.58^**^	0.27^**^	0.26^**^	1

^**^Correlations are significant at p ≤ 0.001.

### 4.1. Existential anxiety and meaning in life

The main objective of the article was to study the interaction between existential anxiety and meaning in life, on one side, and internal resources, on the other side during the COVID-19 outbreak. Given the heterogeneity of the sample, we performed a cluster analysis to identify homogenous groups within the sample based on the overall scores on existential anxiety (EAQ) and meaning in life (MLQ). Cluster analysis was performed using SPSS version 24. Following Milligan's (1980) recommended technique, we first used a hierarchical clustering method that allowed us to visualize how the data may be clustered together. Squared Euclidean distances were used to measure similarity. From this analysis, by examining the dendrogram, we generated a solution with four clusters and conducted k-means clustering with average linkage between groups. Table 3 highlights the characteristics of each cluster—the number of allocated cases, and mean values for the two variables included in the analysis.

**Table 3:** Characteristics of the four-cluster solution.

**Cluster**	**Number of cases included**	**% of cases included**	**EAQ mean value**	**MLQ mean value**	**Name of the cluster**
1	149	33%	3.04 (↑)	4.43 (↓)	Reactive
2	115	25.5%	1.96 (↓)	3.81 (↓)	Superficial
3	58	12.9%	3.03 (↑)	5.85 (↑)	Preoccupied
4	127	28.2%	1.85 (↓)	5.74 (↑)	Dedicated

(↑) = high levels, (↓) = low levels, EAQ, Existential Anxiety; MLQ, Meaning in life.

The first cluster has the highest number of participants (149, 33% of all participants in the study). This cluster includes participants that have above-mean scores for existential anxiety and below-mean scores for meaning in life. We labeled this cluster as *Reactive*. The respondents in this group tend to manifest reactivity in relation to COVID-19 as their existential anxiety is rather high but they do lack a strong presence of meaning in life that could buffer their emotional reaction. The second cluster, labeled as *Superficial*, includes 115 participants (25.5.%) with very low scores on EAQ and below-mean scores on MLQ. This group of participants is characterized by a low activation of existential anxiety and a weak meaning in life, which suggest that these participants tend to rarely concern about death, life, and its purpose. The third cluster includes 58 participants (12.9%) with above-mean scores for both EAQ and MLQ. We labeled this group of respondents as *Preoccupied*. These participants seem to have a strong sense of meaning in life but, at the same time, they are deeply preoccupied with death, fate, meaningless, or condemnation. The fourth cluster includes 127 participants (28.2%) with low existential anxiety and a strong meaning in life. These participants seem to be the *Dedicated* ones who are searching for or have found a strong purpose in their life during the COVID-19 pandemic but are not emotionally overwhelmed by anxious concerns. When testing the intercorrelation between each cluster and exposure to SARS-CoV-2 infection within the social network, no significant association was found (Pearson's chi-square = 8.86, *p* = 0.45).

### 4.2. Well-being and internal resources within each cluster

The second and third research questions of our study referred to identifying significant differences in people's activation of internal resources in relation to existential anxiety and meaning in life. To test this, we performed an analysis of variance between the four clusters for well-being, creativity, playfulness, and personal meaning, followed by *post-hoc* comparisons to show the significant differences. The four groups of respondents—the Reactive, Superficial, Preoccupied, and Dedicated—significantly differ on each tested internal resource (Table 4).

**Table 4:** Differences in well-being, creativity, playfulness and personal meaning between clusters.

**Variables**	**C1—Reactive**		**C2—Superficial**		**C3—Preoccupied**		**C4—Dedicated**		**F**	**η^2^**
	**M**	**SD**	**M**	**SD**	**M**	**SD**	**M**	**SD**		
**Well-being**	4.85	0.70	5.38	0.80	5.11	0.64	5.63	0.56	31.28^**^	0.17
**Creativity**	3.42	0.72	3.46	0.69	3.73	0.58	3.69	0.63	5.40^**^	0.03
**Playfulness**	3.39	0.92	3.47	0.94	3.53	0.83	3.76	0.91	4.00^*^	0.02
**PMP**	4.72	0.65	4.84	0.87	5.33	0.75	5.52	0.65	32.97^**^	0.18

^**^Correlations are significant at *p* ≤ 0.001, ^*^Correlations are significant at *p* ≤ 0.05; PMP, Personal meaning.

**Figure 1:**
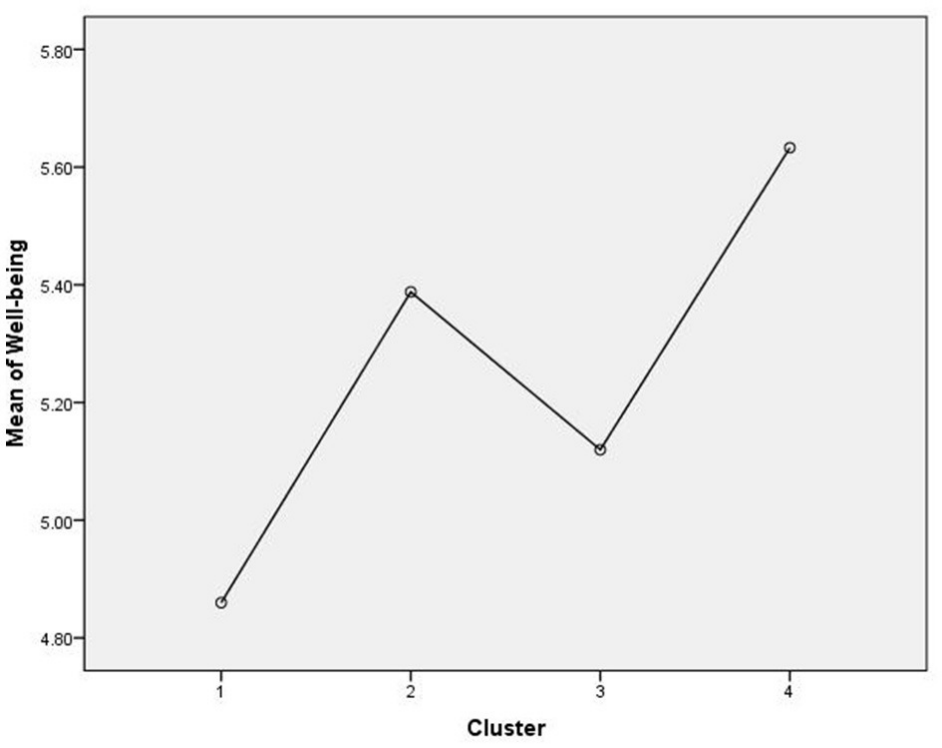
Differences in well-being between the four clusters.

Regarding well-being, we identified a significant difference between the groups [*F*_(3, 445)_ = 31.28, *p* ≤ 0.001]. The Games–Howell test for multiple comparisons showed that the mean value of well-being was significantly higher for C4—Dedicated than for C1—Reactive [*p* ≤ 0.001, 95% C.I. = (0.57, 0.97)], for C2—Superficial [*p* = 0.036, 95% C.I. = (0.01, 0.47)], and for C3—Preoccupied [*p* ≤ 0.001, 95% C.I. = (0.25, 0.76)] (Figure 1). C2—Superficial have higher level of well-being than C1—Reactive [*p* ≤ 0.001, 95% C.I. = (0.28, 0.77)]. As the results show, participants high in existential anxiety and with a medium or low sense of meaning seem to be most affected in their well-being during adverse situations such as the pandemic.

**Figure 2:**
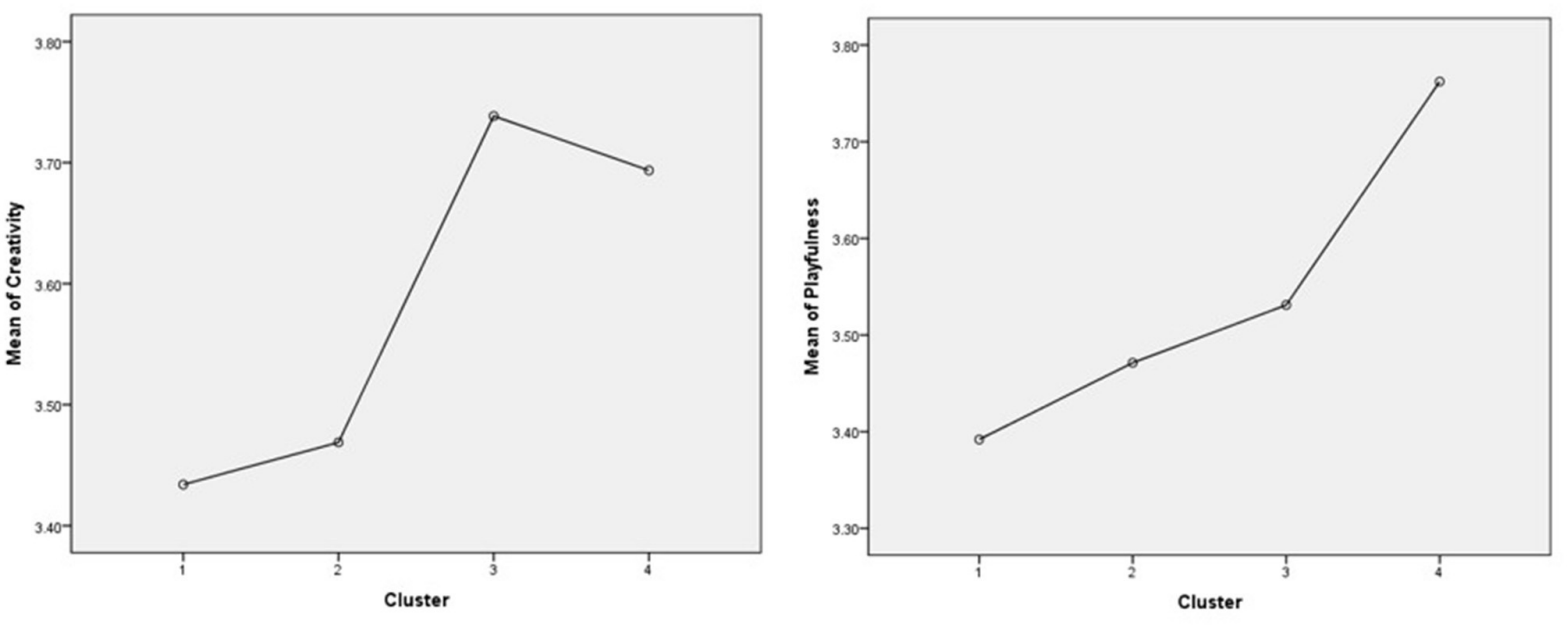
Differences in creativity **(left)** and playfulness **(right)** levels between clusters.

Furthermore, the relationship between the identified clusters and the internal resources showed that people with high existential anxiety and low meaning in life lack creativity and playfulness as assets to cope better in times of disruption, while those with low existential anxiety and high meaning in life have higher levels of creativity and playfulness [for creativity *F*_(3, 445)_ = 5.40, *p* = 0.001; for playfulness *F*_(3, 445)_ = 4.02, *p* = 0.008] (Figure 2). The Games–Howell test for multiple comparisons found that the mean value of creativity was significantly different between C3 and C1 [*p* = 0.012, 95% C.I. = (0.05, 0.55)], C3 and C2 [*p* = 0.41, 95% C.I. = (0.01, 0.53)], respectively, C4 and C1 [*p* = 0.009, 95% C.I. = (0.04, 0.47)], C4 and C2 [*p* = 0.46, 95% C.I. = (0.01, 0.44)]. The Preoccupied cluster and the Dedicated one had the highest levels of creativity, which confirm that people better cope with adversity when they can find meaning in those times and have internal resources which facilitate functional coping. Playfulness showed a significant difference only between Reactive and Dedicated participants [*p* = 0.005, 95% C.I. = (−0.65, −0.08)], but the pattern seems consistent with the idea that less existential anxiety and higher meaning in life is associated with playfulness as a habitual behavior pattern and attitude (Figure 2).

**Figure 3:**
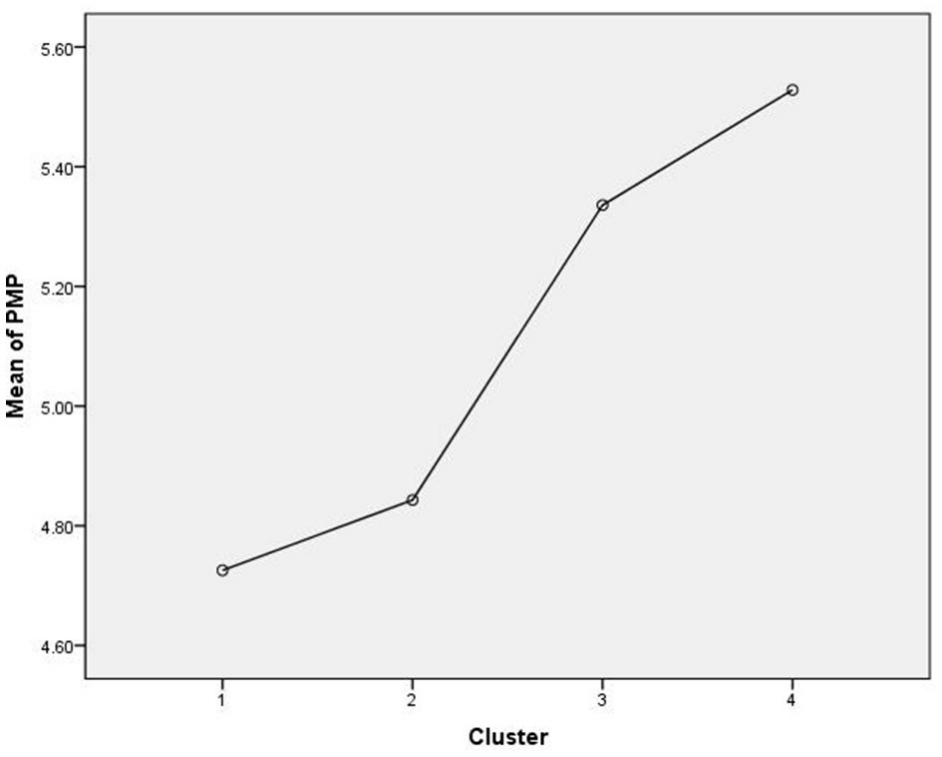
Differences in personal meaning profile between clusters.

Finally, we tested the differences for personal meaning profile which is a comprehensive measure of the meaning in life, implying seven sources of meaning: Achievement, Relationship, Religion, Self-transcendence, Self-acceptance, Intimacy, and Fair treatment. When analyzed, we identified a similar pattern as for the other individual resources. There were significant differences between the groups [*F*_(3, 445)_ = 32.97, *p* ≤ 0.001]. The Games–Howell test for multiple comparisons showed that higher scores were reported in the Dedicated and Preoccupied groups and lower in the Superficial and Preoccupied groups. There were no significant differences between C4 and C3 (*p* = 0.34), and between C1 and C2 (*p* = 0.63) (Figure 3). These results show that finding meaning in different areas of life or from different sources is associated with existential concerns about life and its meaning and it may be a strong resource in coping with traumatic events.

### 4.3. Changes in social media and substances consumption

A final set of analyses was performed to test the association between the four clusters and the consumption of social media, online gaming, and substances during the pandemic. As previously mentioned, these variables were measured on nominal scales, with participants reporting increases or decreases in their habitual use of social media or substances since the beginning of the pandemic. We used Pearson's chi-squared to test the relationships.

**Figure 4:**
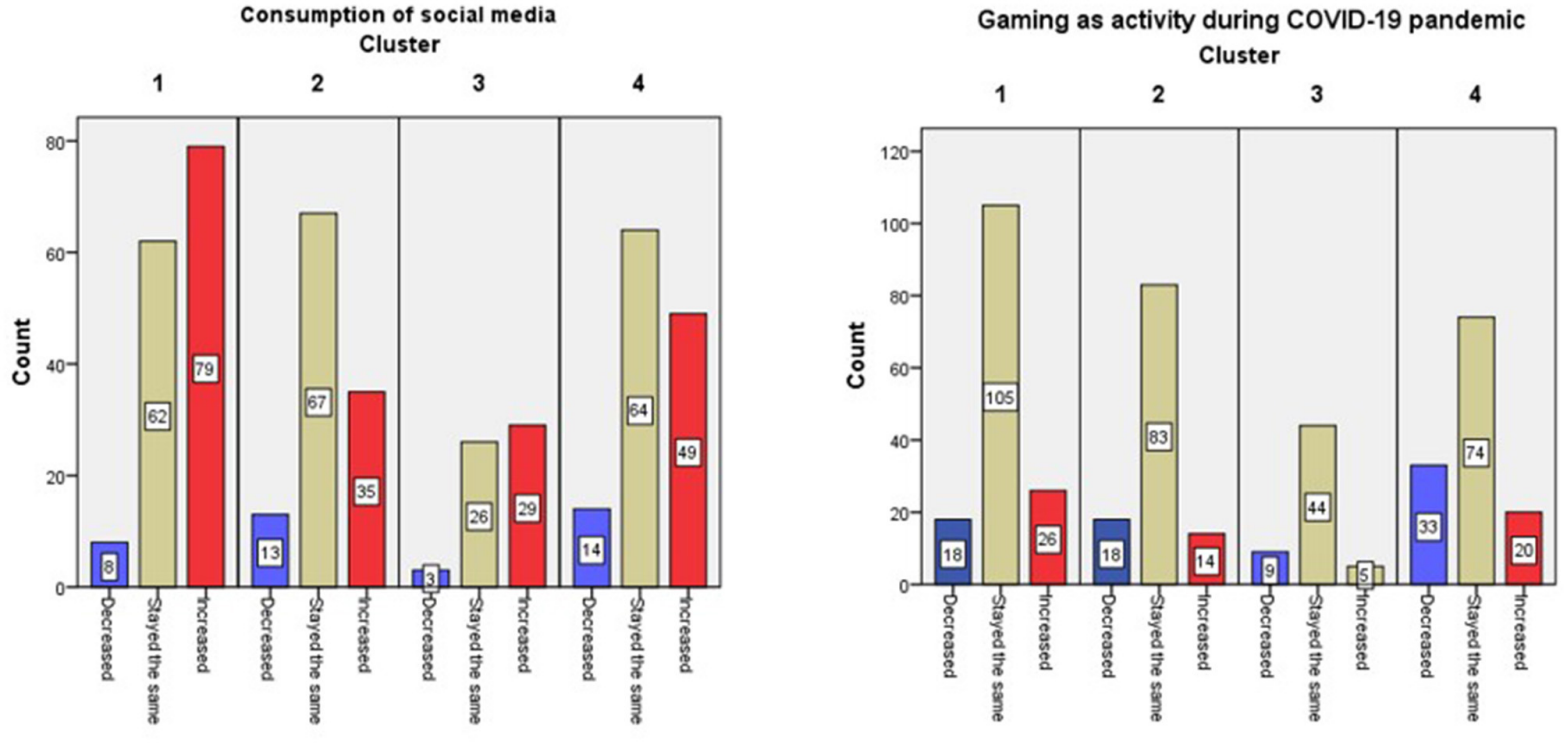
Changes in social media use **(left)** and gaming **(right)** among four clusters.

The results showed a significant relationship between clusters and social media consumption [χ^2^_(6, N = 149)_ = 17.43, *p* = 0.008]. C1 cluster—Reactive included the most participants who reported an increase in their consumption of social media (Figure 4). Online and video gaming also showed significant differences in occurrence among the four groups [χ^2^_(6, N = 149)_ = 13.69, *p* = 0.03]. Cluster 4—Dedicated included the larger number of participants who decreased their gaming activity during the pandemic, while most participants in the other groups declared that time allocated to gaming stayed the same (Figure 4).

No significant differences were found between the groups in terms of alcohol consumption or caffeine consumption [χ^2^_(6, N = 149)_ = 17.20, *p* > 0.05 for alcohol and χ^2^_(6, N = 149)_ = 16.64, *p* > 0.05 for caffeine].

## 5. Discussion

Our study investigated the association between meaning in life as a resource in the face of the aversive context imposed by the COVID-19 pandemic and existential anxiety sprouted by the same global event. Four clusters resulted from the combination of the two dimensions: Reactive (low MLQ and high EAQ), Superficial (low MLQ and low EAQ), Preoccupied (high MLQ and high EAQ), and Dedicated (high MLQ and low EAQ). As Schnell and Becker (2006) suggest, meaning and meaningless crises are not a continuum, but rather two independent dimensions, that were also separated in types by Damásio and Koller (2015). Our study is in line with their finding: The Reactive cluster is similar to the so-called crises category, the Superficial cluster is a mirror of the indifference category, the Preoccupied cluster is similar to existential conflict, and the Dedicated clusters are those with meaningful lives. As will be discussed later, intervention can be built to improve meaning and consecutively well-being.

When tested, data about the number of infections per country or proximity with (in terms of knowing about) people who tested positive or died of COVID-19 did not generate any significant differences among clusters. One explanation might be that data was collected in May—June 2020 and not many casualties appeared by then, many respondents had at that time few situations of knowing people getting infected or dying. Adapting to a crisis is a gradual process and is difficult to obtain relevant information through cross-sectional data collection. A similar result was mentioned by Karataş et al. (2021), in the Turkish population: The presence of the coronavirus infection did not predict life satisfaction.

Identifying types of people depending on their level of existential anxiety and meaning in life was not the only goal. An attempt to identify behavior changes since the lockdown revealed that social media consumption increased in the Reactive cluster, while video gaming decreased significantly in the Dedicated cluster. Alcohol and caffeine intake did not significantly differ across the types. Coping styles during the COVID-19 pandemic were investigated by others and passive or avoidant coping styles were recognized in approximately one-third of the respondents (Fu et al., 2020), or more (Ames-Guerrero et al., 2021), both coping styles being associated with lower levels of psychological well-being (Tuason et al., 2021; Kavčič et al., 2022).

The results on well-being are in line with the body of literature which links meaning in life with well-being but also brings some new insights. Lower levels of well-being were found in the Reactive and Preoccupied clusters, the first being described by low levels of meaning in life, while the second one has both elevated meaning and elevated anxiety. The higher levels of well-being were associated with the Dedicated cluster (also high the in meaning in life), but also elevated well-being was found in the Superficial cluster described by low meaning and low anxiety. Similar results, with positive associations between well-being and meaning in life, were confirmed repeatedly in the literature (Dezutter et al., 2014; García-Alandete, 2015). The authors also mentioned better adjustment and lesser maladaptive profile in people with higher meaning in life, analogous with better inner resources such as creativity and playfulness in our study. Meaning in life was a positive predictor for life satisfaction during the COVID-19 pandemic (Karataş et al., 2021) and also a safeguard for distress inflicted by the coronavirus spread in Australian respondents (Humphrey and Vari, 2021). Pre-pandemic meaning in life had a protective role for depression and anxiety measured peri-pandemic but was found to decrease during the difficult 2020 spring and summer months (Seidel et al., 2023). A direct negative relation was also found between both meaning in life and life satisfaction and fear of COVID-19, MiL mediating the relationship between basic hope and anxiety generated by fear of infection (Trzebiński et al., 2020). Existential anxiety, in light of the EPP model of suffering (Van Tongeren and Showalter Van Tongeren, 2021) activates internal resources in order to overcome distress and negative psychological effects, which translates into higher meaning and higher well-being by the end of the process.

The presence of high levels of well-being in the Superficial type might be explained by a tendency to ignore or neglect reality and to act more for the personal and immediate benefit, which, in the long run, decreases meaning in life but serves to satisfy hedonic tendencies. This finding is in line with Popovic's (2002) view of meaning as having width, depth, length, and temporal dimensions. On the other hand, Bøe et al. (2019) plead for turning attention to “nothings” that matter. They explain the value of superficial, non-sensical, and unidentifiable happenings in our lives, which might lack meaning, but nevertheless are important for the everyday living of normal people.

The relationship between the categories identified and the internal resources showed that people with high existential anxiety and low meaning in life lack creativity and playfulness as assets to cope better in times of disruption. On the other hand, those with low existential anxiety and high meaning in life have higher levels of creativity and playfulness. Positive engagement in the hostile world scenario (as the pandemic might be perceived) is associated with higher well-being and higher meaning in life, while negative engagement exacerbates worries and loneliness (Bergman et al., 2021).

## 6. Conclusion

Some practical implications could be derived from the results. The clear associations between internal resources and meaning in life, although not clarified in terms of causality, highlight the importance of using strength-based intervention as contributors to well-being, more specifically creativity (considered here as general human potential) and humor (as part of playful personality) (Proyer et al., 2013). To elicit meaning, stimulate creativity, or guide people in finding purpose are all instruments to promote psychological and mental health (Steger and Park, 2012). Wong and Bowers (2018) see meaning as having a pivotal role in cultivating psychological well-being, but only with the provision of embracing the dark part of the human condition on earth.

There are some limitations of the present study. First, no analysis included demographic data such as sex, age, education, and marital status. These variables are important factors that can moderate the results given the fact that well-being, for example, is a dynamic construct that varies with age (Ryff and Keyes, 1995; Damásio and Koller, 2015). Older respondents, married people, and women tend to have higher scores in meaning, while younger people have higher scores in well-being. Participants in this study were a heterogeneous group, both geographic and cultural, and in terms of the impact of COVID-19 infection in the early months of spreading.

Several constructs are multifaceted, and no differences were investigated among them: Eudaimonic well-being has six dimensions; meaning in life has two components: the presence of meaning and searching for meaning (Steger et al., 2011). Our main interest was to identify patterns in order to better understand associations among types, and with respect to the view upon meaning in life as a schema-like mechanism, we translate the findings as proof that those higher in meaning are prone to select positive information from life experiences and use them to increase well-being.

Another limitation was that data were collected one-time only, at the beginning of the pandemic, when lockdown was in place and little knowledge about what would be to come could be foreseen. A one-time measure of variables might also be a weakness because the meaning in life and well-being are known to fluctuate and are flexible constructs (Steger and Kashdan, 2013).

Apart from creativity and playfulness, no other personality traits were measured, and there is evidence that personality is important (Schnell and Becker, 2006; Lavigne et al., 2013). Moreover, negative psychological impacts (such as depression, stress, and negative emotions) other than existential anxiety did not enter our analysis. Future investigation might consider the Big Five model or even self-esteem and self-efficacy.

Another comment worth mentioning is that people tend to report above-average levels in both meaning in life and well-being, and it is rare in normal populations to identify truly low scores for these variables (Damásio and Koller, 2015). It is not possible to estimate the levels of the variables measured in our respondents before the pandemic, and a repeated-measured design might be of interest in the future when investigating people's reactions to an existential crisis.

## Data availability statement

The raw data supporting the conclusions of this article will be made available by the authors, without undue reservation.

## Ethics statement

The studies involving humans were approved by Faculty of Psychology and Education Sciences, Transilvania University of Braşov. The studies were conducted in accordance with the local legislation and institutional requirements. The participants provided their written informed consent to participate in this study.

## Author contributions

LTD and CT equally contributed to the conceptualization and design of the study. LTD organized the database, performed the statistical analysis, and wrote the first draft of the Introduction section. CT wrote the first draft of the Methods section and the Result section. All authors contributed to the manuscript revision, read, and approved the submitted version.
